# Common rs7138803 variant of *FAIM2* and obesity in Han Chinese

**DOI:** 10.1186/1471-2261-13-56

**Published:** 2013-08-08

**Authors:** Cong Li, Xueping Qiu, Na Yang, Jiajia Gao, Yuan Rong, Chenling Xiong, Fang Zheng

**Affiliations:** 1 Center for Gene Diagnose, Zhongnan Hospital of Wuhan University, Wuhan, China

**Keywords:** FAIM2, Single nucleotide polymorphism, Obesity susceptibility

## Abstract

**Background:**

Obesity causes severe healthcare problem worldwide leading to numerous diseases, such as cardiovascular diseases and diabetes mellitus. Previous Genome-Wide Association Study (GWAS) identified an association between a single nucleotide polymorphism (SNP) rs7138803, on chromosome 12q13 and obesity in European Caucasians. Since the genetic architecture governing the obesity may vary among different populations, we investigate the variant rs7138803 in Chinese population to find out whether it is associated with obesity.

**Methods:**

A population-based cohort association study was carried out using the High Resolution Melt (HRM) method with 1851 participants. The association between rs7138803 genotypes and body mass index (BMI) was modeled with a general linear model, and a case–control study for the association between rs7138803 genotypes and obesity was performed using Pearson’s χ^2^ test. There was no indication of a deviation from Hardy-Weinberg equilibrium (HWE p value = 0.51) in our sample.

**Results:**

No association was detected between SNP rs7138803 and BMI in our Chinese Han population with a *P* value of 0.51. SNP rs7138803 was found to be not associated with common forms of obesity after adjusting for age and sex in the Chinese population. SNP rs7138803 was not associated with other obesity related traits, including T2DM, hypertension, lipid profiles, and ischemic stroke.

**Conclusion:**

Our data suggest that the rs7138803 exerts no significant effect on obesity in Chinese Han population. Larger cohorts may be more appropriate to detect an effect of this SNP on common obesity.

## Background

Obesity has become a growing health problem which affects more than 400 million people worldwide [1]. Obesity predisposes to type 2 diabetes mellitus (T2DM), cardiovascular diseases, dyslipidemia, hypertension, sleep apnea and some forms of cancer [2]. A number of studies have pointed toward a strong hereditary component of obesity, identifying genetic variants that influence human BMI will advance our understanding of how environmental and genetic factors interplay together to lead to obesity. GWAS offers the opportunity to investigate a number of complex traits. In the recent years, huge progresses based on the large scale association have been made on the susceptibility genes linked to obesity. Gene variants have been reproducibly confirmed to affect the common forms of obesity [3-10].

However, not all of the reported associations could be replicated in other ethnic cohorts even if the association was carried out in large-scale samples in the original report [11]. Therefore, cross-ethnic validation of the association is crucial to establish the role of susceptibility genes. Large scale GWAS identified an association between common variant rs7138803, between *BCDIN3D*, *FAIM2* gene of 12q13 and obesity in over 30,000 adult individuals of European Caucasians [7,12,13]. After the initial finding, this observation was validated in multiple Caucasian populations and Asian populations [14-18]. However, the reports from Chinese population were controversial, as several reports indicated the same effect of rs7138803 in Chinese population as that in Caucasian populations [19-21], but some reports indicated that rs7138803 was not associated with BMI or obesity in Chinese [22,23]. Furthermore, effect evaluation in population-based European ethnics also implicated that common variants of *FAIM2* gene may contribute to overall body size rather than just adiposity [24].

We look up the biological function of the neighboring gene *FAIM2*, and note that *FAIM2* (Fas apoptotic inhibitory molecule 2,also known as LFG) encodes a protein that inhibits the apoptotic signal uniquely from Fas receptor, also called Apo1 [25]. It is speculated that widely distributed *FAIM2* possibly plays an important role in adipocyte apoptosis. Furthermore *FAIM2* is remarkably highly expressed in the hippocampus and may therefore influence the neural development of feeding. Since a number of studies on the association of rs7138803 with obesity and T2DM have been performed, and controversial results were reported among different research groups [19,26]. It turns out to be necessary to study the exact relationship between rs7138803 and obesity-related diseases such as hypertension, T2DM and ischemic stroke.

The present study carried out both qualitative and quantitative analysis of obesity-related metabolic traits in mainland Chinese Han individuals. The study was trying to expand the association of the *FAIM2* SNP rs7138803 with obesity previously identified in multiple cohorts of European descents to a Chinese Han population, and to further provide the evidence of obesity susceptibility of the *FAIM2* locus to multiple obesity-related traits.

## Methods

### Study population

The study subjects were from a subsample of WD-CAD and Beijing stroke cohort (BJSC). WD-CAD was enrolled as an epidemiological study evaluating factors relating to cardiovascular diseases, including 11,568 Chinese Han population (both CAD patients and healthy controls) at five hospitals in Wuhan and Dalian; BJSC was recruited mainly from 2 hospitals of the city of Beijing for the case–control study of stroke. SNP rs7138803 was genotyped in totally 1,851 subjects, all subjects are of the ethnic Han origin according to medical files, 543 subjects were recruited from WD-CAD study and 1308 subjects were from BJSC study. We defined obesity as BMI ≥ 27.5 kg/m^2^, based on the World Health Organization recommended obesity criteria for Asians [27]; Case–control study in 242 obese cases and 469 normal-weight controls (18.5 ≤ BMI ≤ 23.0 kg/m^2^) was performed to test the obesity association based on the criteria. Smoking and drinking history were collected by direct interviews or from medical files. The study was approved by ethics committee of Zhongnan Hospital of Wuhan University and met the declaration of Helsinki.

### Genotyping of SNP rs7138803

A Rotor-Gene™ 6000 High Resolution Melt system (Corbett Life Science, Concorde, NSW, Australia) was used for all genotyping assays of SNP rs7138803. Genotyping was performed in 25 μL of standard PCR volume containing 1 μL of LC Green dye, 5 pmol of each primer, 25 ng of genomic DNA, 2.5 μL of 10× PCR buffer with 1.5 mM MgCL_2_, 5 mmol deoxynucleotide triphosphates and 1 U of Taq polymerase. Two positive controls for each genotype (A/A, A/G and G/G) were included in each run. A total of 24 samples were randomly selected for verification of genotyping results using direct DNA sequence analysis.

### Diagnosis criteria

All subjects had detailed medical history taken and measurements including height, weight, clinical blood pressure and lipid profiles. Height and body weight were measured in light indoor clothes and without shoes, BMI was calculated as weight/height^2^ (kg/m^2^). Hypertension was defined as clinical blood pressure (BP) higher than 140/90 mm Hg, patients classified as previously hypertensive if they had a personal history of hypertension (ie., BP >140/90 mm Hg) or taking antihypertensive medication, were also included, secondary hypertension was excluded according to diagnosis of the doctor [28]. For the measurement of clinical blood pressure, 3 readings were made, using a conventional mercury sphygmomanometer with an appropriate size cuff, on the nondominant arm. Systolic blood pressure (SBP) was defined as the pressure level at which the first of 2 regular Korotkoff sounds were heard. Diastolic blood pressure (DBP) was defined as pressure level of the last of these rhythmic sounds. Clinical blood pressure was defined as the mean of the second and third blood pressure readings [28]. Pulse pressure (PP) was calculated as the difference between the SBP and DBP.

Lipid profiles of all participants were available on medical files. Total cholesterol (TC), high-density lipoprotein cholesterol (HDL-C) and triglyceride (TG) concentrations were analyzed by an Olympus AU2700 Clinical Analyzer after overnight fasting, and low-density lipoprotein cholesterol (LDL-C) concentration were calculated using the standard Friedewald formula. Atherosclerosis index (AI) was calculated by the formula: atherosclerosis index = (serum TC − HDL-C) / HDL-C. Diagnosis of ischemic stroke was based on the World Health Organization (WHO) criteria by a panel of clinical neurologists [29]. All patients with ischemic stroke had MRI or CT performed.

### Statistical analysis

SNP rs7138803 genotypes were tested for Hardy-Weinberg equilibrium in the population using PLINK v1.06. Allelic and genotypic association of rs7138803 with obesity was assessed using Pearson’s 2 × 2 and 2 × 3 contingency table χ^2^ test (SPSS, version 13.0). Statistical significance was defined as a p value < 0.05. Odds ratios (ORs) and 95% confidence intervals (CIs) were estimated using the χ2 test (SPSS, version 13.0). The statistical power of the study was calculated using the PGA software [30]. Multivariate analysis was performed by incorporating age and sex as covariates using multivariate logistic/linear regression (SAS, version 9.0). Empirical p values were determined using the PLINK v1.06 program with 100,000 Monte Carlo simulations. Linear regression was used to estimate the per allele effect of rs7138803 on BMI.

## Results

### Descriptive characteristics

Clinical characteristics of all participants in this study were shown in Table 1. High efficiency of genotyping was obtained with the success and concordance rates of > 94% and > 99%, respectively, the minor allele A frequency of SNP rs7138803 in our sample was 28.8%. In 1851 samples, prevalence of obesity (BMI ≥ 27.5 kg/m^2^) were 13.1%. Average BMI in our study was 24.47, which was lower than that of western origin. The cardiovascular traits of WD-CAD and BJSC cohorts were similar, except for the proportion of CAD, T2DM and ischemic stroke. We performed all associations by pooling WD-CAD and BJSC cohorts together in this study.

**Table 1:** Clinical characteristics of WD-CAD and BJSC cohorts

	**WD-CAD**	**BJSC**	**Combine**
n	543	1308	1851
Male/female	301/242	782/526	1083/768
Age (mean ± SD)^1^	61.1 ± 10.5	62.0 ± 10.6	61.7 ± 10.6
Obesity	80 (14.7%)	162 (12.4%)	242 (13.1%)
Hypertension (%)	89 (16.4%)	267 (20.4%)	356 (19.2%)
CAD (%)	39 (7.2%)	8 (0.6%)	47 (2.5%)
T2DM (%)	188 (34.6%)	29 (2.2%)	217 (11.7%)
TC	4.28 ± 1.05	5.17 ± 1.25	4.87 ± 1.26
TG	1.71 ± 1.07	1.77 ± 1.24	1.75 ± 1.18
HDL	1.25 ± 0.30	1.27 ± 0.46	1.26 ± 0.39
LDL	2.48 ± 0.77	3.21 ± 1.08	2.77 ± 0.97
Ischemic stroke(%)	43 (7.9%)	1042 (79.7%)	1085 (58.6%)

^1^ age at the enrollment for both WD-CAD and BJSC cohort.

### Linkage disequilibrium map construction

**Figure 1:**
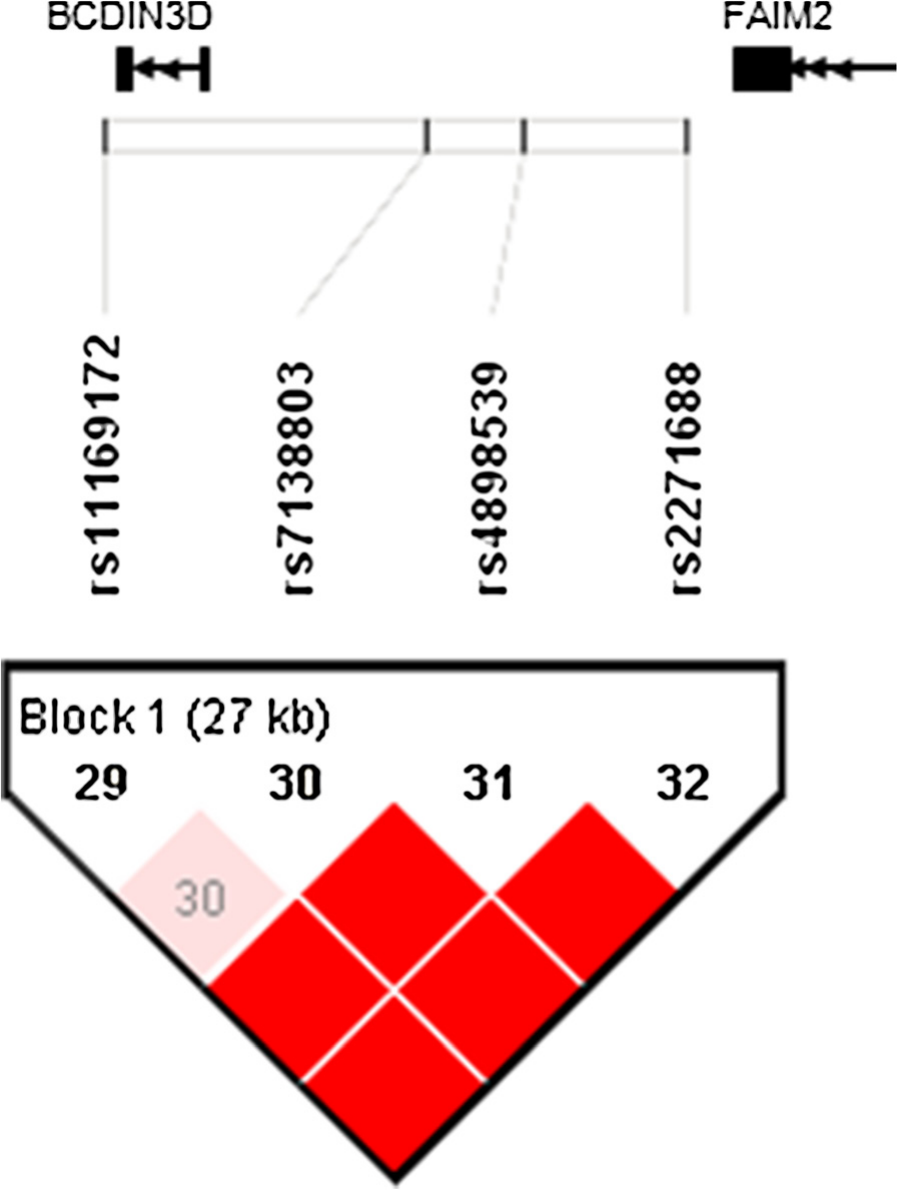
**Overview of linkage disequilibrium (LD) of the 40 kb region around rs7138803 of *FAIM2/BCDIN3D*.** The LD structure around the SNP rs7138803 was derived from the genotyping data of the HapMap for the Chinese population (http://www.hapmap.org, phase 2, HapMap-HCB). The pairwise correlation between SNPs was measured as D’ and a red diamond without a number refers to D’ = 100. Exons are marked with black boxes. The direction of ZFHX3 transcriptions was to the left as marked by the arrows.

We constructed a linkage disequilibrium (LD) map for a 40 kb genomic region around rs7138803 of *FAIM2/BCDIN3D* using the genotyping data for the Chinese samples from the HapMap database (http://www.hapmap.org, phase 2, HapMap-HCB). SNP rs7138803 located in the intergenic region between *FAIM2* and *BCDIN3D*, the LD between SNP rs7138803 and the 2 genes seemed strong according to the LD map (Figure 1).

### Association between rs7138803 with obesity and obesity-related traits

A statistical power analysis was carried out to calculate the sample size in the study to evaluate the association between rs7138803 and obesity; our combined WD-CAD and BJSC cohort had a 74.5% statistical power to evaluate the association, the OR and the risk allele frequency was in accordance with previously published results [23]. No significant deviation from Hardy–Weinberg equilibrium (HWE) was observed in our population (p = 0.51). Minor A-allele was supposed to exert a risk on obesity according to the reports. However, no significant association was observed in the case–control association (242 obese cases VS 469 normal-weight controls) after multivariate logistic regression for age and sex (p = 0.37, OR = 1.11, 95% CI of 0.88–1.41), no significant association between the rs7138803 variant and T2DM was demonstrated (p = 0.86, OR = 0.97, 95% CI of 0.72–1.32) (Table 2). The association with BMI was not significant in our total 1,851 subjects using linear regression either (p = 0.51). When the 1,851 subjects were divided into a male group and a female group, still no association existed in both groups with p values of 0.43 and 0.55, respectively (Table 3).

**Table 2:** Obesity, T2DM and ischemic stroke association with SNP rs7138803 in our study

		**Obesity**				**T2DM**				**Ischemic stroke**			
		**No. of**	**No. of**	**p-value**^**1**^	**OR (95% CI)**^**1**^	**No. of**	**No. of**	**p-value**^**1**^	**OR (95% CI)**^**1**^	**No. of**	**No. of**	**p-value**^**1**^	**OR (95% CI)**^**1**^
		**cases**	**controls**			**cases**	**controls**			**cases**	**controls**		
Allele frequencies	A	153	275	0.37	1.11 (0.88–1.41)	137	598	0.86	0.97 (0.72–1.32)	619	136	0.59	1.06 (0.85–1.32)
	G	331	663			297	1260			1465	342		
Genotype frequencies	AA	29	34	0.08	/	26	113	0.96	/	84	16	0.77	/
	AG	95	207			85	372			451	104		
	GG	118	228			106	444			507	119		

^1^ Multivariate logistic regression models were used to estimate p-values, odds ratios and 95% confidence intervals.

**Table 3:** Association of rs7138803 A allele with obesity-related quantitative traits

**Phenotype**	**CAF**^**1**^	**β**^**2**^	**p*-obs***^**3**^	**p*-adj***^**4**^
BMI	0.29	0.053	0.69	0.51
BMI in male	0.29	0.076	0.64	0.43
BMI in female	0.28	0.028	0.89	0.55
TC	0.30	0.009	0.21	0.24
TG	0.29	0.003	0.18	0.19
HDL-C	0.29	−0.0003	0.99	0.99
LDL-C	0.31	−0.090	0.64	0.63
Systolic blood pressure	0.29	0.012	0.60	0.65
Diastolic blood pressure	0.29	0.003	0.38	0.39
Pulse pressure	0.29	0.095	0.17	0.17
Atherosclerosis index	0.28	−0.010	0.23	0.29

^1^*CAF* coded allele (the allele for which effect was estimated) frequency;

^2^ β, per coded allele effect;

^3^ p*-obs*: observed uncorrected p values;

^4^ p-*adj*: Adjusted p values were obtained using multivariate linear regression for age and sex.

In the combined cohort, fasting levels of serum total cholesterol, triglyceride and HDL, LDL cholesterol were available. Since there was possible significant correlation between BMI and lipid profiles, we also investigated the potential association of total cholesterol (TC), triglyceride (TG), HDL-cholesterol and LDL-cholesterol with rs7138803, but no significant association was demonstrated after carrying out quantitative association analysis in our study (Table 3). Isolated diastolic/systolic-diastolic hypertension is more prevalent in hypertensive obese individuals [31,32]. we therefore investigated the possible association between hypertension and rs7138803, SBP and DBP were not associated with rs7138803 (p = 0.65 and p = 0.39), neither were the PP and AI (Table 3).

Since hypertension strongly predisposes to stroke [33], we tried to assess whether variant rs7138803 was associated with ischemic stroke in our cohorts. We took patients (n = 1,042) diagnosed as ischemic stroke as cases, healthy individuals (n = 239) from WD-CAD as controls. Our data indicated no significant association between ischemic stroke and rs7138803 (Table 2).

## Discussion

A GWAS identified a SNP rs7138803 on chromosome 12q13 for common obesity in mainly European-derived populations. Since then multi-ethnic studies demonstrated that the SNP rs7138803 was associated with obesity, and with T2DM in some ethnics. However, some reports provided evidence against the association [22], it is still in debate whether the SNP rs7138803 exerts effects on obesity and related clinical traits, especially in Chinese population. Here we extended the study to measurements of BMI, blood pressure and metabolic related traits in Chinese Han population.

We failed to replicate the previous finding that rs7138803 A-allele was responsible for the increasing risk of obesity. Therefore, we hypothesized that rs7138803 might increase the risk of obesity by interacting with environmental factors, such as physical activity levels [17], or other genetic factors. Furthermore, the majority of participants in our analysis had cardio-vascular risk factors and stroke background, the population differences between previous studies and ours may lead to the negative finding. In addition, the small effect size which leads to false negative findings might explain the lack of association. However, our findings must be further replicated in larger samples using populations from the same origin. Considerable evidence indicated that obesity contributed to the development of T2DM, and plasma lipid levels, particularly TG and HDL [34,35]. However, the SNP showed no evidence of significant association with any of these traits in our study.

The sample size of HapMap-HCB covering *FAIM2*/*BCDIN3D* region was small, therefore, the LD values around the *FAIM2*/*BCDIN3D* region could not be confirmed. SNP rs7138803 variant could be in LD with another SNP, which was not studied in the present analysis, but could contribute to obesity and related traits. In the populations of European ancestry, the minor allele A of rs7138803 was the risk allele for obesity and had a frequency of about 43.2%. In contrast, the frequencies of rs7138803 A allele in the HapMap-HCB and our study samples were only 32.2% and 28.8%, respectively. The dramatic difference of allele frequencies between the populations of European ancestry and Chinese populations may be a cause for lack of association between rs7138803 and obesity.

There are growing evidence that obesity and weight gain clearly have a negative impact on blood pressure [36]. The exact mechanism is still obscure but hyperinsulinemia, insulin resistance, sodium retention and cardiac output may be involved in obesity-related hypertension. Weight gain increases blood pressure even over a period of several weeks, and weight loss produces a corresponding decrease in blood pressure, it has also been found that every kilogram excess body weight loss is associated with decreases of 0.33 and 0.43 mmHg in SBP and DBP respectively [37]. In our study, there was no association between rs7138803 and blood pressure.

Since hypertension is the major cause of ischemic stroke, over the range of 115/75 to 185/115 mm Hg, each 20-mm Hg elevation in SBP (or 10-mm Hg elevation in DBP) roughly doubles the risk of stroke death [37]. The mechanism that BP rise leads to ischemic stroke onset is unknown and questions remain to be clarified, genetic factors are assumed to play an important role in the development of ischemic stroke. Therefore, we further assessed whether rs7138803 A allele conferred a risk for ischemic stroke, a total of 1042 patients with ischemic stroke were selected as case, hemorrhagic stroke and cardioembolic stroke were excluded, 239 healthy individuals without CAD or T2DM from WD-CAD were chosen as controls, our results suggested that rs7138803 was not a risk factor for ischemic stroke in the Chinese population. However, this may be still partly due to the small sample size of controls from WD-CAD population.

## Conclusions

We found no association between SNP rs7138803 on chromosome 12q13 and obesity in the mainland Chinese Han population, and no significant association between rs7138803 and T2DM was found either. Together, these results indicate that the future research has to fine-map the association of genes localized in the locus 12q13 of *FAIM2* to shed light on the mechanism for the pathogenesis of obese related disease. And also consider and explore the possibility of the existence of other gene variants that are in LD with the SNP studied in this analysis, and which may indeed be involved in the determination of complex metabolic diseases and related traits.

## Abbreviations

GWAS: Genome-wide association study; HRM: High resolution melt; HWE: Hardy-Weinberg equilibrium; BMI: Body mass index; CAD: Coronary artery disease; TC: Total cholesterol; TG: Triglyceride; HDL-C: High-density lipoprotein cholesterol; LDL-C: Low-density lipoprotein cholesterol; SNP: Single nucleotide polymorphism; T2DM: Type 2 diabetes mellitus; SBP: Systolic blood pressure; DBP: Diastolic blood pressure; AI: Atherosclerosis index; PP: Pulse pressure.

## Competing interests

The authors declare that they have no competing interests.

## Authors’ contributions

CL and FZ designed and performed this study and drafted the manuscript. XQ was responsible for running the HRM assays, CL and NY performed the statistical analysis, JG performed part of the sequencing experiments, YR and CX was responsible for clinical patient data and diagnosis of study population, FZ supervised the recruitment of the patients and compliance with Institutional ethical procedures, FZ took responsibility for the project. All authors have read and approved the final manuscript.

## Pre-publication history

The pre-publication history for this paper can be accessed here:

http://www.biomedcentral.com/1471-2261/13/56/prepub
